# Pain and pain management following manual versus robotic assisted unicondylar knee arthroplasty

**DOI:** 10.1016/j.jor.2025.07.008

**Published:** 2025-07-09

**Authors:** Felix C. Oettl, Aaron I. Weinblatt, Yu-Fen Chiu, Gwo-Chin Lee, Stephen Lyman, Brian Chalmers, Matthew Austin, Alejandro Gonzalez Della Valle

**Affiliations:** a Hospital for Special Surgery, New York, NY, USA; b Department of Orthopedic Surgery, Balgrist University Hospital, University of Zürich, Zurich, Switzerland

**Keywords:** Robotic-assisted unicondylar knee arthroplasty, Unicondylar knee arthroplasty, Postoperative pain, Opioid utilization, Length of hospital stay

## Abstract

**Aims:**

The adoption of robotic assistance for unicondylar knee arthroplasty (UKA) is increasing, driven by reports of improved implant positioning. However, its impact on short-term patient outcomes remains debated. This study aimed to compare postoperative pain, opioid consumption, and length of hospital stay between manual (maUKA) and robotic-assisted (raUKA) procedures in a large, real-world cohort.

**Materials and methods:**

We retrospectively identified 1369 opioid-naïve patients undergoing medial, unilateral UKA at a single institution between 2019 and 2023 (417 manual, 952 robotic). We collected data on Numeric Pain Rating Scale (NRS) scores, opioid consumption in morphine milligram equivalents (MMEs), and length of hospital stay. Multivariable linear regression was used to compare outcomes while controlling for patient-level confounders.

**Results:**

After multivariable adjustment, we found no statistically significant difference between the manual and robotic groups in length of hospital stay (p = 0.6206) or total 90-day opioid consumption. Patients in the raUKA group reported slightly higher pain scores at the first postoperative measurement (Estimate −0.7, p < 0.001); however, no significant differences were observed in average, minimum, or maximum in-hospital pain scores. There was no significant difference in total inpatient opioid consumption.

**Conclusion:**

In this large single-institution analysis, robotic assistance was not associated with improvements in postoperative pain, opioid use, or length of hospital stay compared to the manual technique. These findings suggest that potential benefits of robotic UKA related to implant accuracy may not translate to improved short-term clinical outcomes, a crucial consideration in the context of technology acquisition and healthcare costs.

## 1 Introduction

In recent years there has been a growing emphasis on reducing the risks associated with prescription opioids, which include opioid use disorder and opioid-related mortality.^1^, ^2^, ^3^ A significant contributor to the opioid prescription burden are orthopedic surgical procedures, which account for nearly 10 % of all opioid prescriptions in the United States.^4^, ^5^, ^6^, ^7^

Postoperative pain is one of many factors influencing length of stay, complications, and poor patient satisfaction.^8^ Pain control following knee arthroplasty is multifactorial and appropriate pain management remains a key consideration for both surgeons and patients.

Robotic arm-assisted UKA (raUKA) has emerged as a technological advancement, with studies consistently demonstrating more accurate and reproducible implant placement compared to manual techniques^9^, ^10^, ^11^, ^12^ It is hypothesized that this precision, potentially leading to minimized bone resection and soft tissue trauma, may contribute to diminished postoperative pain and faster recovery.^9^^,^^13^ However, the existing literature presents a conflicted view on these clinical benefits. While some prospective studies, such as those by Blyth et al. and Kayani et al. have suggested lower pain scores and shorter hospital stays following raUKA, these findings are not universally reported..^11^^,^^14^ This discrepancy highlights the need for further investigation, particularly in large, real-world settings where patient and procedural variability may differ from controlled trials.

Therefore, the primary objective of this study was to determine if robotic assistance has a significant effect on postoperative pain, as measured by the Numeric Rating Scale (NRS), and postoperative opioid consumption, measured both in-hospital and up to 90 days post-operatively. Our secondary objectives were to evaluate the impact on the length of hospitalization for patients undergoing UKA. We hypothesized that, within our institution's standardized care pathways, there would be no significant difference in these short-term clinical outcomes between manual and robotic-assisted techniques.

## 2 Material and methods

Following institutional review board approval, a retrospective observational cohort study was conducted in patients undergoing UKR at a single academic institution between January 2019 and June 2023.

We identified 1369 patients having undergone medial UKA between January 1, 2019 and June 30, 2023 of which 417 were manual UKA (maUKA) and 952 were raUKA (Table 1). We chose this timeframe, because our institution's opioid management protocols were consistent throughout this period.

**Table 1:** Patient characteristics.

		All		Manual		Robotic		
		N =	1369	N =	417	N =	952	
		**Mean**	**SD**	**Mean**	**Std**	**Mean**	**SD**	**P-value**
Age		64.5	9.9	63.9	9.2	64.7	10.1	0.119
BMI		29.2	5.0	29.3	5.0	29.1	4.9	0.586

		**N**	**%**	**N**	**%**	**N**	**%**	**P-value**

Sex								0.099
	Women	627	45.8	177	42.4	450	47.3	
CCI								0.450
	0	993	72.5	295	70.7	698	73.3	
	1	269	19.6	90	21.6	179	18.8	
	2	72	5.3	24	5.8	48	5	
	3+	35	2.6	8	1.9	27	2.8	
ASA Level								0.546
	1	48	3.5	17	4.1	31	3.3	
	2	1158	84.6	356	85.4	802	84.2	
	3	156	11.4	43	10.3	113	11.9	
Race								0.338
	Asian	46	3.4	16	3.8	30	3.2	
	Black or African American	65	4.7	22	5.3	43	4.5	
	Native Hawaiian or Other Pacific Islander	2	0.1	1	0.2	1	0.1	
	Other	90	6.6	36	8.6	54	5.7	
	White or Caucasian	1156	84.4	339	81.3	817	85.8	
Ethnicity								0.125
	Hispanic or Latino	93	6.8	37	8.9	56	5.9	
Married								0.454
	Yes	1026	74.9	307	73.6	719	75.5	
Smoking status								0.919
	Current	12	0.9	3	0.7	9	0.9	
	Former	462	33.7	141	33.8	321	33.7	
	Never	893	65.2	272	65.2	621	65.2	
Insurance								0.042
	Other	25	1.8	13	3.1	12	1.3	
	Public	483	35.3	138	33.1	345	36.2	
	Private	861	62.9	266	63.8	595	62.5	
Nerve block								0.0003
	N/A	17	1.2	9	2.2	8	0.8	
	Adductor Canal	172	12.6	75	18	97	10.2	
	Femoral	63	4.6	15	3.6	48	5	
	IPACK	4	0.3	0	0	4	0.4	
	IPACK + AC	1113	81.3	318	76.3	795	83.5	
Regional anesthesia								0.201
		1331	97.2	409	98.1	922	96.8	

Demographic characteristics of patients undergoing manual and robotic unicondylar knee arthroplasty are presented. Continuous variables are reported as mean (± standard deviation, SD), and categorical variables as count (percentage). P-values represent statistical significance between manual and robotic unicondylar knee arthroplasty groups.Abbreviations: ASA, American Society of Anesthesiologists; CCI, Charlson Comorbidity Index; IPACK, Infiltration betweenthe Popliteal Artery and Capsule of the Knee, AC, Adductor Canal.

## 3 The outcomes assessed were grouped into several categories

Post-operative pain assessed using a NRS based on the following criteria: first score documented after surgery, lowest score during hospitalization, highest score during hospitalization, and mean score calculated from all recordings during hospitalization.

Inpatient dosing of non-opioid analgesics and antiemetics was quantified as the mean hourly dose administered during the entire hospitalization for the following medications: acetaminophen, ondansetron, celecoxib, meloxicam, ketorolac, gabapentin, and pregabalin.

Inpatient opioid utilization was quantified in oral morphine milligram equivalents (MME, 7.5 MMEs equal 5 mg oxycodone pill^15^) and split into: total MME administered during hospitalization, mean hourly MME during hospitalization, total MME after surgery during hospitalization and mean hourly postoperative MME. MMEs prescribed at hospital discharge was determined from opioid prescriptions in the discharge medication records. Total outpatient MMEs utilized within 90 days after discharge were calculated from recorded opioid prescription fills. Total MME the patient consumed was calculated based on inpatient and prescribed MMEs between the day of surgery and 90 days postoperatively. Days to final opioid prescription refill defined as the number of days between discharge and the final opioid prescription refill within the 90-day post-discharge period.

Length of hospital stay (LOS) defined as the time elapsed between admission and discharge reported in hours. Post-anesthesia care unit (PACU) length of stay defined as the duration between PACU admission and discharge to the ward reported in hours.

## 4 Institutional pain-management protocol

Patients routinely underwent spinal anesthesia and periarticular anesthetic blocks during the procedure (Table 1). Postoperatively, the regimen consisted of scheduled acetaminophen 650–1000 mg every 6 h, meloxicam 7.5–15 mg daily, oxycodone 5–10 mg every 4–6 h as needed, and intravenous hydromorphone for breakthrough pain. Physical therapy with weight-bearing as tolerated commenced on the day of surgery or first postoperative day. Upon discharge, patients continued the same oral medication regimen as an outpatient, with physical therapy ongoing. Discharge narcotic prescriptions varied due to factors such as patient preference, tolerance, allergies, and narcotic use history. For opioid refills, patients contacted the postoperative nurse practitioner or surgeon's office directly. Prescribers were mandated to consult the statewide prescription monitoring program to screen for potential abuse before dispensing controlled substances. Refills were provided if no evidence of abuse existed, with quantities determined by the prescriber's assessment of the patient's needs.

### 4.1 Statistical analysis

A Smirnov Kolgomorov test was performed to assess the assumpition of a normal distribution for all continuous outcome variables. Descriptive statistics are reported as means (standard deviation [SD]) and median (interquartile range [IQR]). Categorial variables are reported as frequency and percentages. Inferential analyses of continuous outcome variables relied on independent samples t-tests or their non-parametric equivalent. Categorical variables were assessed using chi-square tests or Fisher's exact test depending on expected cell sizes.

A multivariable regression analysis was performed to assess the independent effects of robot versus manual UKA. This analysis controlled for age, sex, BMI, Charlson Comorbidity Index (CCI), ASA Class, race, marital status, smoking status, insurance type, and use of nerve block. Significance was defined as p < 0.05 Statistical analyses were performed using SAS 9.4 (SAS Institute Inc., Cary, NC) and Rstudio February 1, 5042 (RStudio, Inc., Boston, MA).

## 5 Results

Patients undergoing raUKA reported statistically significant higher pain scores on the first measurement taken postoperatively (1.51 vs 0.83; p < 0.0001), a difference that was preserved after risk adjustment (Estimate −0.70; p < 0.0001) (Table 2, Table 3). However, this initial difference was small and did not extend to other pain metrics. There were no significant differences in the lowest, highest, or average NRS pain scores reported during hospitalization between the two groups after multivariable analysis (Table 3).

**Table 2:** Continuous outcomes.

	Manual (n = 417)		Robotic (n = 952)		
	Mean (SD)	Median (IQR)	Mean (SD)	Median (IQR)	P-value
Length of Stay (hours)	22.8 (19.5)	11 (10–31)	20.4 (16.3)	11.5 (9–30)	0.094
Time spent in PACU (hours)	5.27 (2.45)	5 (4–6)	4.97 (2.2)	5 (4–6)	0.011
avg hourly dose of acetaminophen	71.57 (83.77)	37.04 (0–142.86)	69.89 (89.26)	37.04 (0–125)	0.470
avg hourly dose of ondansetron	0.05 (0.16)	0 (0-0)	0.04 (0.16)	0 (0-0)	0.105
avg hourly dose of celecoxib	0.45 (2.19)	0 (0-0)	0.05 (0.74)	0 (0-0)	<0.0001
avg hourly dose of meloxicam	0.11 (0.21)	0 (0-0)	0.11 (0.22)	0 (0-0)	0.427
avg hourly dose of ketorolac	0.96 (1.48)	0 (0–1.43)	0.74 (1.16)	0 (0–0.94)	0.087
avg hourly dose of gabapentin	1.2 (12.22)	0 (0-0)	0.46 (4.99)	0 (0-0)	0.362
avg hourly dose of pregabalin	0.02 (0.48)	0 (0-0)	0.02 (0.24)	0 (0-0)	0.353
lowest NRS pain	0.05 (0.57)	0 (0-0)	0.09 (0.53)	0 (0-0)	0.024
highest NRS pain	5.92 (2.5)	7 (4–7)	5.91 (2.64)	7 (4–8)	0.724
avg NRS pain	2.24 (1.44)	2.14 (1.2–3.25)	2.35 (1.54)	2.31 (1.13–3.5)	0.278
first NRS pain^#^	0.83 (2.22)	0 (0-0)	1.51 (2.87)	0 (0-0)	<0.0001
MME 90 days excluding inpatient	180.41 (386.99)	0 (0–210)	157.11 (371.45)	0 (0–210)	0.298
number of opioid refills	0.79 (1.61)	0 (0–1)	0.68 (1.47)	0 (0–1)	0.263
days to last opioid refill	9.41 (18.02)	0 (0–12)	8.42 (17.99)	0 (0–7)	0.222
MME - Total 90 Da y	284.67 (517.31)	0 (0–510)	246.64 (481.91)	0 (0-0)	0.305
MME Total Inpatient	47.42 (58.64)	31.5 (15–60)	40.01 (42.23)	30 (15–52.5	0.143
MME Inpatient Postop	34.26 (55.67)	15 (7.5–40)	27.24 (35.97)	15 (7.5–35)	0.266
MME - Discharge	290.38 (85.43)	315 (300–315)	283.94 (81.98)	315	0.456
				266.25–315)	
MME/hr Inpatient Postop	2.24 (2.29)	1.69 (0.31–30.33)	2.28 (2.32)	1.73 (0.45–3.33)	0.856
MME/hr Total Inpatient	2.26 (1.99)	1.74 (0.83–3.33)	2.14 (1.78)	1.7 (0.83–3.13)	0.564

Descriptive statistics and univariate analysis comparing manual and robotic arm assisted unicondylar knee arthroplasty are displayed. Continuous variables are expressed as mean (± standard deviation, SD) or median (interquartile range, IQR) as appropriate. P-values represent the significance of differences between the manual and robotic UKA groups.Abbreviations: PACU, post-anesthesia care unit; LOS, length of stay; NRS, numeric rating scale; MME, morphine milligram equivalents.

**Table 3:** Regression analysis.

maUKA vs raUKA	N = 1369			
Outcome variables:	Estimate	95 % CI		P-Value
Length of stay (hours)	0.50	−1.49	2.50	0.6206
Time spent in PACU (hours)	0.19	−0.09	0.48	0.1817
avg hourly dose of acetaminophen	0.01	−0.01	0.03	0.2882
avg hourly dose of ondansetron	0.43	0.26	0.60	<0.0001
avg hourly dose of celecoxib	−0.03	−0.06	0.00	0.0253
avg hourly dose of meloxicam	0.19	0.03	0.34	0.0183
avg hourly dose of ketorolac	0.61	−0.38	1.60	0.2269
avg hourly dose of Gabapentin	0.00	−0.04	0.04	0.9546
lowest numeric rating scale pain	−0.05	−0.10	0.01	0.096
highest numeric rating scale pain	−0.14	−0.46	0.18	0.3842
avg numeric rating scale pain	−0.10	−0.28	0.08	0.2759
first numeric rating scale pain	−0.70	−1.04	−0.35	<0.0001
MME – last refill within 90 Days	16.50	−29.40	62.40	0.4812
MME - Total 90 Da y	29.33	−31.70	90.37	0.3464
MME Total Inpatient	3.95	−1.87	9.77	0.1838
MME Inpatient Postop	3.45	−1.78	8.69	0.1966
MME - Discharge	11.08	1.29	20.88	0.0267
MME/hr Inpatient Postop	0.01	−0.27	0.29	0.9462
MME/hr Total Inpatient	0.18	−0.04	0.40	0.1074

The estimates of outcome variables from a multivariable regression model, with 95 % confidence intervals (CI) and associated p-values are presented. Positive estimates indicate higher values in the robotic arm assisted unicondylar knee arthroplasty compared to manual unicondylar knee arthroplasty group, while negative estimates reflect lower values in robotic arm assisted unicondylar knee arthroplasty. The analysis adjusts for relevant covariates, and statistically significant differences are highlighted with p-values less than 0.05.Abbreviations: PACU, post-anesthesia care unit; LOS, length of stay; NRS, numeric rating scale; MME, morphine milligram equivalents.

There is no statistical significant difference in opioid consumption between maUKA and raUKA in univariate analysis. However, a trend is shown with patients undergoing maUKA receiving more opioids during and after hospitalization (Table 2). An independent association was observed with maUKA patients receiving significantly more MMEs at discharge than raUKA patients (Estimate 11.1, p < 0.05). This difference is not statistically significant during hospitalization or 90-day follow-up (Table 3).

There was no statistically significant difference in the length of hospital stay between the manual and robotic UKA groups in either univariate (22.8 vs 20.4 h; p = 0.094) or multivariate analysis (p = 0.6206) (Table 3). While time spent in the PACU was significantly shorter for the raUKA group in univariate analysis (5.27 vs 4.97 h, p = 0.011), this difference did not remain significant after adjusting for confounders (p = 0.1817).

None of the categorical variables showed a difference between maUKA and raUKA.

## 6 Discussion

In an era of value-based healthcare, the adoption of new, more expensive technologies must be justified by clear clinical benefits. While robotic systems for UKA have demonstrated improved radiographic accuracy, their effect on patient-centered outcomes such as pain, opioid use, and recovery speed remains a subject of debate.^16^ The principal finding of our large, single-institution retrospective study is the lack of a significant association between robotic assistance and improvements in length of hospital stay, postoperative pain levels, or opioid consumption up to 90 days post-surgery. This result challenges the assumption that superior implant precision automatically translates into a better short-term patient experience and carries important implications for hospital and patient decision-making.

After correcting for known confounding variables, we did detect significantly greater MME at discharge in patients undergoing maUKA. We did not detect a difference in other in-hospital or post-discharge opioid consumption patterns between maUKA and raUKA. We did see a significant difference favoring the maUKA group in pain immediately after surgery, which we hypothesize might be explained by the additional pins required for raUKA. Nonetheless minimum, maximum and average pain did not show a significant difference between the groups at all other recorded timepoints postoperatively. After risk adjustment, hourly non-opioid medication consumption showed significantly greater ketorolac, ondansetron and meloxicam consumption in maUKA with higher celecoxib consumption in raUKA. Hospitalization duration, as well as time spent in the PACU, were similar for both groups after risk adjustment.

A prospective study conducted by Blyth et al.^11^ involving 139 patients undergoing medial UKA randomized to either manual conventional cutting instruments or haptic robotic assistance, demonstrated significant reductions in pain during the initial two postoperative months following robotic UKA. The authors observed that, from the first postoperative day until week 8 after UKA, the median pain scores for the robotic group were 55.4 % lower than those of the manual surgery group (p = 0.040). In contrast, to our findings as average and minimum pain showed no advantage for the raUKA group (lowest: Estimate −0.05, 95 %CI -0.1– 0.01, p = 0.096; average: Estimate −0.1, 95 % CI -0.28–0.08, p = 0.279).

Kayani et al.^14^ conducted a prospective consecutive cohort study involving 146 patients who underwent medial UKA using either conventional jig-based instrumentation or robotic arm assistance. The study reported that robotic arm-assisted UKA was associated with reduced postoperative pain (p < 0.001), decreased opioid requirements (p < 0.001), and shorter length of hospitalization (p < 0.001). While these outcomes are encouraging, the study did not specify if perioperative pain management protocols were consistent between the groups. If the protocols were indeed similar, the reduced opioid requirements observed in their study warrant highlighting, particularly given the Blyth et al. study,^11^ Crizer et al. study^17^ and our study did not find a significant advantage for raUKA in postoperative pain levels or opioid consumption. Our recorded average length of stay is substantially shorter for both groups compared to those included by Kayani (maUKA 71.1 h vs 22.79 h; raUKA 42.5 h vs 20.41 h) and the authors suggest that the difference in length of stay and opioid consumption might be attributed to the consecutive enrollment of patients, starting with maUKA, leading to raUKA gaining an advantage from improvements in postoperative management.

Our findings stand in contrast to some prospective studies, as Kayani et al. and Blyth et al. reported that raUKA was associated with reduced pain and a shorter length of hospitalization.^11^^,^^14^ However, our study, one of the largest to date, did not replicate these advantages. This discrepancy may be explained by several factors, especially as our institution has highly developed, standardized rapid recovery protocols, which may create a “ceiling effect” for any additional benefits from robotic technology. Notably, the average length of stay in our cohort for both manual (22.8 h) and robotic (20.4 h) groups was substantially shorter than that reported by Kayani et al. (71.1 h for manual, 42.5 h for robotic), suggesting our baseline is already highly optimized.

This study has several limitations inherent to its retrospective design. The most significant of these is the potential for unmeasured confounding variables despite our use of multivariable adjustment. Chief among these are surgeon-level variations; we did not control for individual surgeons' standard intraoperative and postoperative protocols. Factors such as soft tissue handling, the administration of local anesthetic injections, postoperative weight-bearing restrictions, and the choice of prescribed analgesic medications were left to the clinical judgment of the attending surgeon. While including cases from multiple high-volume surgeons enhances the “real-world” generalizability of our findings, it also introduces this procedural heterogeneity. Methodological limitations also warrant discussion. The sample size, while substantial, was smaller than the cohort we initially intended to analyze from 2016 onwards. This impacts statistical power and introduces the classic challenge of multiple comparisons. Without formal correction, statistically significant findings could be spurious (a Type I error), yet applying stringent corrections could mask a true, albeit small, clinical effect (a Type II error). Furthermore, our analysis was constrained by the scope of the outcome measures. We did not assess patient-reported functional outcomes (e.g., KOOS, Jr., LEFS), which are crucial for a comprehensive understanding of recovery. Additionally, it is important to highlight that our post-discharge opioid data reflects morphine milligram equivalents (MMEs) *prescribed*, not necessarily *consumed*, and we cannot accurately capture the latter. It is also possible that the outcome measures we employed lack the sensitivity to differentiate more nuanced degrees of clinical benefit between the two techniques. Collectively, these factors underscore the need for a cautious interpretation of our results. Larger prospective studies, ideally randomized controlled trials with standardized protocols, are necessary to validate these findings. Such trials would also be better suited to explore the influence of surgeon experience and would benefit from including a broader range of functional outcome measures.

In conclusion, this large-scale analysis found no evidence that robotic assistance in UKA provides a clinical advantage over manual techniques with respect to postoperative pain, opioid consumption, or length of stay. While the value of robotic systems in achieving radiographic targets is well-established, these benefits did not translate into a measurably better short-term clinical course for patients in our cohort. These null findings are significant, suggesting that the routine adoption of this expensive technology may not be justified by the short-term patient-reported outcomes measured here.

## CRediT authorship contribution statement

**Felix C. Oettl:** All listed authors have contributed substantially to this work, performed primary manuscript preparation, Editing and final manuscript preparation was performed, All authors read and approved the final manuscript. **Aaron I. Weinblatt:** All listed authors have contributed substantially to this work, developed the idea for the present study, performed primary manuscript preparation, Editing and final manuscript preparation was performed, All authors read and approved the final manuscript. **Yu-Fen Chiu:** All listed authors have contributed substantially to this work, were responsible for methodology and statistical analysis, All authors read and approved the final manuscript. **Gwo-Chin Lee:** All listed authors have contributed substantially to this work, Editing and final manuscript preparation was performed, All authors read and approved the final manuscript. **Stephen Lyman:** All listed authors have contributed substantially to this work, were responsible for methodology and statistical analysis, All authors read and approved the final manuscript. **Brian Chalmers:** All listed authors have contributed substantially to this work, Editing and final manuscript preparation was performed, All authors read and approved the final manuscript. **Matthew Austin:** All listed authors have contributed substantially to this work, Editing and final manuscript preparation was performed, All authors read and approved the final manuscript. **Alejandro Gonzalez Della Valle:** developed the idea for the present study, performed primary manuscript preparation, Editing and final manuscript preparation was performed, All authors read and approved the final manuscript.

## Guardian/patient's consent

A waiver of informed consent was granted in accordance with IRB guidelines, given that the study involves the use of de-identified data, ensuring patient confidentiality and privacy.

## Ethical approval

This study has been reviewed and approved by the Institutional Review Board (IRB) under Expedited Review Category #5. The approval was granted on January 31, 2024, and will expire on January 3, 2027 (Study# 2024–0196). All necessary privacy and confidentiality measures, including the assignment of unique study numbers, will be strictly adhered to.

Informed consent: A waiver of informed consent was granted in accordance with IRB guidelines, given that the study involves the use of de-identified data, ensuring patient confidentiality and privacy.

## Funding

This study was partially funded by the generous donation of the Peterson Foundation.

## Conflict of interest

SL is a paid consultant for CellSource. GCL receives royalties from Corin and Conformis and has stock or stock options in Corin and Parvizi Surgical Innovations. AGDV receives royalties from OrthoDeveloment, is a paid consultant for Johnson& Johnson, Link Orthopaedics, Naviswiss and OrthoDevelopment, furthermore he received research support from OrthoSensor.

## Figures and Tables

**Table:** 

CCI	Charlson Comorbidity Index
KOOSJr	Knee Injury and Osteoarthritis Outcome Score, Joint Replacement
LEFS	Lower Extremity Functional Scale
LOS	Length of hospital stay
maUKA	manual UKA
MMEs	morphine milligram equivalents
NRS	Numeric pain rating Scale
PACU	Post-anesthesia care unit
raUKA	Robotic arm-assisted UKA
UKA	unicondylar knee arthroplasty
